# Basalt derived from highly refractory mantle sources during early Izu-Bonin-Mariana arc development

**DOI:** 10.1038/s41467-021-21980-0

**Published:** 2021-03-19

**Authors:** He Li, Richard J. Arculus, Osamu Ishizuka, Rosemary Hickey-Vargas, Gene M. Yogodzinski, Anders McCarthy, Yuki Kusano, Philipp A. Brandl, Ivan P. Savov, Frank J. Tepley, Weidong Sun

**Affiliations:** 1 Center of Deep Sea Research, Institute of Oceanology, Chinese Academy of Sciences, Qingdao, China; 2 Laboratory for Marine Mineral Resources, Qingdao National Laboratory for Marine Science and Technology, Qingdao, China; 3 Center for Ocean Mega-Science, Chinese Academy of Sciences, Qingdao, China; 4 Research School of Earth Sciences, Australian National University, Canberra, Australia; 5 Geological Survey of Japan/AIST, Tsukuba, Ibaraki Japan; 6 Institute for Marine Geodynamics, Japan Agency for Marine-Earth Science and Technology, 2-15 Natsushima-cho, Yokosuka, 237-0061 Japan; 7 Department of Earth & Environment, AHC5-394, Florida International University, Miami, FL USA; 8 Department of Earth & Ocean Sciences, University of South Carolina, Columbia, SC USA; 9 Institute of Earth Sciences, University of Lausanne, Lausanne, Switzerland; 10 GEC, CY Cergy Paris Université, Cergy, F-95 000 France; 11 GEOMAR Helmholtz Centre for Ocean Research Kiel, Kiel, Germany; 12 School of Earth & Environment, University of Leeds, Leeds, UK; 13 College of Earth, Ocean, and Atmospheric Sciences, Oregon State University, Corvallis, OR USA

**Keywords:** Geochemistry, Geology, Petrology

## Abstract

The magmatic character of early subduction zone and arc development is unlike mature systems. Low-Ti-K tholeiitic basalts and boninites dominate the early Izu-Bonin-Mariana (IBM) system. Basalts recovered from the Amami Sankaku Basin (ASB), underlying and located west of the IBM’s oldest remnant arc, erupted at ~49 Ma. This was 3 million years after subduction inception (51-52 Ma) represented by forearc basalt (FAB), at the tipping point between FAB-boninite and typical arc magmatism. We show ASB basalts are low-Ti-K, aluminous spinel-bearing tholeiites, distinct compared to mid-ocean ridge (MOR), backarc basin, island arc or ocean island basalts. Their upper mantle source was hot, reduced, refractory peridotite, indicating prior melt extraction. ASB basalts transferred rapidly from pressures (~0.7-2 GPa) at the plagioclase-spinel peridotite facies boundary to the surface. Vestiges of a polybaric-polythermal mineralogy are preserved in this basalt, and were not obliterated during persistent recharge-mix-tap-fractionate regimes typical of MOR or mature arcs.

## Abstract (web summary)

Magmatism associated with early growth of subduction zones is unlike that of mature island arc systems. Here, the authors find basalts with distinct mineralogical and geochemical characteristics were erupted during this early stage, and derived from extremely refractory, hot mantle sources.

## Introduction

Chains of explosive stratovolcanoes are characteristic of island and continental arcs such as the IBM and Andes, respectively. These chains form a volcanic front typically no closer than 100 km from the nearest trench, and lie ~105 km above the uppermost surface of a subducting lithospheric plate (slab)^1,2^. Basalt-dominated back-arc basins generated by seafloor spreading have formed during intervals in the history of most of the western Pacific arcs. These features have developed over several tens of millions of years. The trace element and isotopic characteristics of arc magmas are distinctive compared with the basalts of mid-ocean ridges (MORB) and those of isolated ocean islands (OIB) such as Hawaii. The distinctiveness derives from the fact that arc magmas have compositions modulated by the involvement of slab-derived fluids and melts, and soluble trace elements entrained therein.

In a steady state, the pressure-temperature-dependent nature of the metamorphic mineral assemblages, and specifically hydrous phase stability that is established in the uppermost portion of a subducted slab, coupled with the thermal structure of the mantle wedge, control the depth at which slab-derived components are delivered to the wedge^3,4^. These factors impose the characteristic dimensions of volcanic front-trench-slab top distances identified above. However, subduction zones and arcs are ephemeral. Many of those currently active in the western Pacific were initiated about 50 Ma ago, possibly accompanying a major change in plate motions of the region^5,6^. The tectonic conditions preceding subduction inception have been hotly debated and attributed to spontaneous and induced modes^7^. The active examples of subduction inception are the Puysegur-Hjort system south of New Zealand^8,9^, and the propagating southeastern portion of the New Hebrides (<2 Ma)^10^. Studies of the IBM system^11,12^ and Central America^13^ have however, established the broad tectonic and temporal framework in and during which the respective arcs were initiated. Prior to the establishment of a (quasi-) steady state in a given subduction zone system, the initial magmatic outputs in nascent arcs are of prime interest for their potential in revealing fundamental characteristics such as the nature of the earliest mantle wedge, temporal changes in the inputs from the subducted slab, depths from which the magmas were derived, and petrological characteristics that serve to identify examples in the geological record.

Boninite magma (high-MgO; low-TiO_2_, low- to intermediate SiO_2_ (>8; <0.5; 53–63 wt%, respectively) sourced from a newly established mantle wedge together with forearc basalt (FAB) have been regarded as the archetypal nascent arc magma types^11,14,15^. Neither of these magma types is derived by partial melting of the slab. For example, hydrous partial melting of very hot, refractory (clinopyroxene-poor harzburgite) mantle sources at relatively low pressures (<2 GPa) is indicated for boninite^14,16^.

**Fig. 1:**
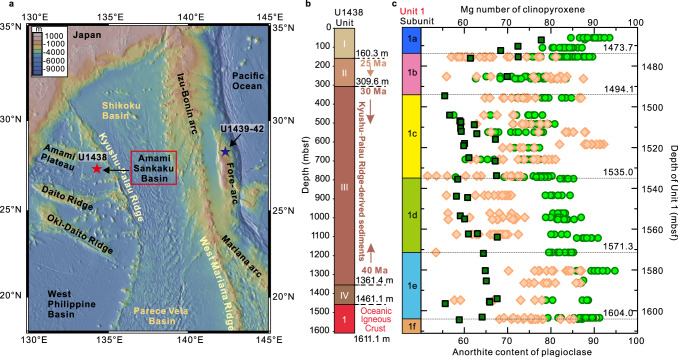
Location of IODP expeditions 351 (U1438) and 352 (U1439-42) drill sites, stratigraphy of U1438, bulk rock and mineral characteristics of Unit 1 at Site U1438. **a** Bathymetric map^65^ showing the location of Amami-Sankaku Basin and Site U1438 (red star) adjacent to the Kyushu-Palau Ridge (remnant arc), and sites U1439-42 (black star; Expedition 352) in the present-day fore-arc. Figure made with GeoMapApp (www.geomapapp.org). **b** Unit thicknesses and ages at Site U1438. Units are coloured for visualisation. **c** subunit information for Unit 1 including Mg number (100∗Mg/(Mg+Fe^2+^)) of clinopyroxene (green circles), anorthite content (An%) of plagioclase (orange diamonds), and bulk rock 100∗Mg/(Mg + ΣFe) (dark green squares) versus depth. Colours of subunits are also used in all other figures (main text and supplementary).

The stratigraphy exposed both along the IBM trench wall and the western flank of the Ogasawara Ridge collectively reveals peridotite succeeded by gabbro, FAB and then boninite^11,15,17,18^. Ages for FAB (and related gabbros) and boninite recovered at these locations by dredging, submersible and at International Ocean Discovery Program (IODP) Sites U1439 and U1442 (Fig. 1), range from 51.9 to 51.3 Ma for FAB, and 51.3 to 50.3 Ma for boninite^19^. Boninite generation along the Ogasawara Ridge occurred between 48 and 45 Ma^18,20,21^. Younger (~45–44 Ma), boninite and 2-pyroxene andesites outcrop in the northern parts of the Chichijima Island group^20^. Tholeiitic basalts and andesitic (calc-alkaline) rocks 44-37 Ma age occur in the Hahajima islands, south of Chichijima^21^.

The Ti/V of both the IBM FAB and boninite is distinctly lower than MORB or the regional back-arc basin basalts (BABB), as a result of prior source mantle depletion in extractable melt, and perhaps of the higher oxidation state of the renewed partial melting process accompanying subduction inception^15,22^. The evidence for slab-derived trace element additions is equivocal in the basalts; enrichments in Rb and U relative to other potentially fluid-mobile and rare earth elements (REEs) can be attributed to seafloor alteration in place rather than derived from the subducted slab.

In 2014, in addition to IODP expedition 352 to the forearc, expedition 351 explored the foundations of the IBM system in the Amami Sankaku Basin (ASB) (Site U1438) (Fig. 1a). Based on seismic evidence, recovery was anticipated of the pre-IBM oceanic basement on which the earliest topographically prominent arc, represented by the Kyushu-Palau Ridge (KPR) chain of stratovolcanoes, is located. However, beneath 1460 m of overlying sediments (Fig. 1b), the expedition penetrated 150 m of basalt that shares much of the geochemical character^23^ with but younger age (49 Ma)^24^ than FAB. Site U1438, located ~60 km southwest of the KPR, is a distal extreme from the current trench of all IBM-related magmatism so far drilled (Fig. 1). At the time of emplacement and assuming no slab rollback, Site U1438 was at least 250 km distal from the position of the current trench after accounting for subsequent backarc spreading in the Shikoku Basin and without allowing for any subduction erosion along the trench^25^. The known distribution of low-Ti-K tholeiitic basalts emplaced in the first few million years of the IBM arc system, is thus extensive, both along- and across the strike of the arc^17^.

In this work, we examine the most important petrological characteristics of the basalt comprising Unit 1 of ASB basalt, and explore the distinctive conditions under which it was generated. Unit 1 basalts comprise low-Ti-K, aluminous spinel-olivine-plagioclase-clinopyroxene-bearing tholeiites derived from hot, reduced, refractory peridotite at pressures ranging from ~0.7 to 2 GPa.

## Results

### Petrology of unit 1 ASB basalt

**Fig. 2:**
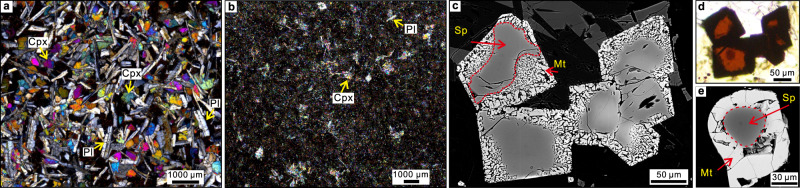
General and detailed photomicrographs of Unit 1 ASB basalts. **a** Medium-grained basalt from subunit 1c (78R3 21–25), including clinopyroxene (Cpx) and plagioclase (Pl) crystals (crossed polars). **b** Fine-grained basalt (84R1 84–87) from subunit 1e, with small crystals of clinopyroxene and plagioclase (crossed polars). **c** Back-scatter electron images of spinel; darker colour within the red dash line is aluminous spinel (Sp); brighter colour outside the red dash line is symplectitic magnetite (Mt). **d** Transmitted light image of the same spinel shown in c, red colour is the Al-rich cores, and opaque boundaries are magnetite. **e** Back-scatter image of spinel, Al-rich core surrounded by magnetite-rich rim outside the red dash line.

On the basis of morphology, chemical composition, and isotopic characteristics, the basalt sheet lavas and pillows comprising Unit 1 have been divided into several subunits (a–f; Fig. 1c)^26,27^. Representative examples in thin-section of the textures and minerals of the subunits are shown in Fig. 2 and Supplementary Fig. 1. Textures range from glassy through fine-grained microcrystalline to medium-grained, phyric to sparsely microphyric, plus some that are intersertal to medium-grained sub-ophitic. The phenocryst and microphenocryst mineralogy comprise plagioclase, clinopyroxene, olivine and spinel. Based on the petrographic relationships and trace element variations in the respective minerals, a crystallisation order of spinel, olivine, plagioclase, and clinopyroxene is inferred. All minerals appear fresh except for olivine, which is mostly pseudomorphed by chlorite and calcite. Some olivine in subunit 1b has a composition of Fo_90–90.5_, which is considerably more Mg-rich than the clinopyroxene (Mg number 80–75) of the same subunit. The groundmasses vary from glassy (average 35% and <85%) to holocrystalline comprising plagioclase, clinopyroxene, and magnetite. One section is moderately vesiculated (30%) but the majority are sparsely to non-vesicular. Many flow contacts exist, some with (altered) glassy margins but most with gradational changes in grain size. Numerous thin (<3 mm) and branching veins filled with calcite, chlorite, and clay minerals are present throughout Unit 1. Logging shows zones of varying redox where the veins also contain either hematite, or pyrite, or magnetite; other sections have blotches and veins of chlorite^26^.

The MgO contents of Unit 1 ASB basalt vary from 13.8 to 6.7 wt% with a mean of 8.6 wt%; the highest values are in subunits 1a and e^26^. The higher values of subunit 1a may be affected by seawater alteration, given elevated Na, K and soluble trace element (Rb, U) abundance characteristics^26^. However, the persistent offset and tracking of the Mg number (100∗Mg/(Mg+Fe^2+^) of the clinopyroxene at higher values than those of the host rock 100∗Mg/(Mg + ΣFe) shown in Fig. 1c for all subunits, is consistent with intrinsically high MgO contents of Unit 1 basalts compared with MORB (mean 7.6 wt%; generally <10 wt%)^28^. The high Cr (≤500 ppm) and Ni (≤350 ppm) of Unit 1 lavas are also consistent with a primitive character. The TiO_2_ contents range from 0.6 to 1.2 wt.% with a mean of 0.9; this is significantly lower than that of MORB (mean = 1.68 wt%)^28^. Some lavas of the high Mg# subunit 1e have K_2_O contents <0.02 wt%^24^, which is an order of magnitude less than the mean for MORB (0.16 wt%^28^). The majority of the lava of Unit 1 can accordingly be classified as low-Ti-K tholeiitic basalts on the basis of their low-Ti-K in combination with total alkalis vs. silica contents^29^.

**Fig. 3:**
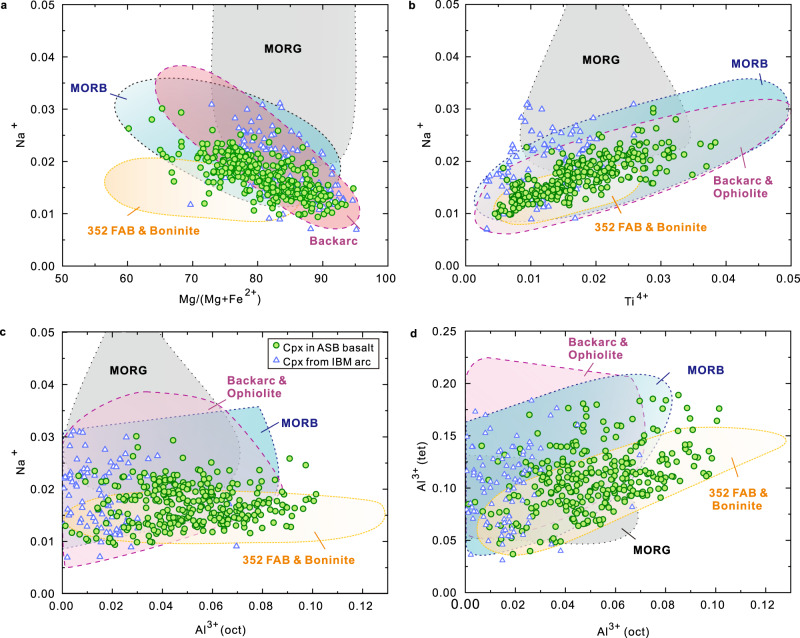
Clinopyroxene compositions in 351 Unit 1 of ASB basalt (green circles) compared with 351 Unit IV (blue triangles) recording the nascent IBM arc^35^ immediately overlying the ASB basalt, mid-ocean ridge basalt and gabbro (MORB/G), 352 FAB and boninite^36^, Oman ophiolite^37^ and backarcs (Data from PetDatabase website). Consistently coloured shading and lettering are used for these specified compositional fields. **a** Na^+^ versus 100∗Mg/(Mg + Fe^2+^) for clinopyroxene in Unit 1 ASB basalt and Unit IV recording the nascent IBM arc^35^, showing compositional overlap compared with clinopyroxene in MORB/G and backarcs, but a contrasted trend compared with FAB and boninite; **b** Na^+^ versus Ti^4+^. **c** Na^+^ versus octahedrally coordinated Al^3+^. **d** Tetrahedrally coordinated versus octahedrally coordinated Al^3+^ (Al^3+^ (tet) and Al^3+^ (oct) respectively). All cations calculated on the basis of 6 oxygen anions. References for data sources used for clinopyroxene in MORB/G are in the Supplementary Material. Oman ophiolite and backarc data mostly overlap, scatter plots are in Supplementary Fig. 2.

The presence of (micro)phenocrystic clinopyroxene (Fig. 2) in all Unit 1 ASB basalts is remarkable compared with the typical truancy of this phase as a phenocryst in primitive [high 100∗Mg/(Mg + ΣFe)] MORB^30^. Clinopyroxene is unequivocally involved in the establishment of the overall major and trace element variation patterns in the global MORB compositional array^31^, and is a major component of gabbros recovered from MOR sections (e.g.,^32^). The clinopyroxene in Unit 1 basalts spans a range from diopside to augite according to the IMA classification^33^, with Mg numbers up to 95 (Figs. 1c, 3a). Major and trace element analyses are presented in Supplementary Table 1, and additional major elements only in Supplementary Table 2. Selected grouped binary compositional plots of these analyses are presented in Fig. 3. Data for clinopyroxene in individual subunits and Unit III^34^ are shown in Supplementary Fig. 2. Unit III comprises volcaniclastic-rich sediments of Eocene-Oligocene-age derived from the KPR^34^ (data in Supplementary Table 3). In Fig. 3, comparative data are displayed for: clinopyroxene grains from within the oldest sediments recording nascent IBM arc (Unit IV; Eocene age^35^) overlying Unit 1 ASB basalt; MORB and MOR gabbros (MORG; references in Supplementary Material); references in Supplementary Material); 352 FAB and boninite^36^, Oman ophiolite^37^ and backarc basalts (Data from PetDatabase website) (Fig. 3, Supplementary Fig. 2).

Some features of the comparative clinopyroxene compositional plots are clear: (1) Na is negatively correlated with Mg number, and positively correlated with Ti in both unit 1, III, and IV (Fig. 3a, b, Supplementary Fig. 2); (2) Na is much lower at high Mg number than those in MORB/G. (Fig. 3a); (3) Unit 1 clinopyroxene is generally distinctly more aluminous in both tetrahedrally- and octahedrally coordinated Al than in clinopyroxene of Unit III or IV and extends to more aluminous compositions than MORB/G. Subunits a and e have the highest Mg number decreasing through f and d to subunits b and c. In contrast, clinopyroxene for all subunits spans a similar range of Al (tetrahedrally- and octahedrally coordinated).

**Fig. 4:**
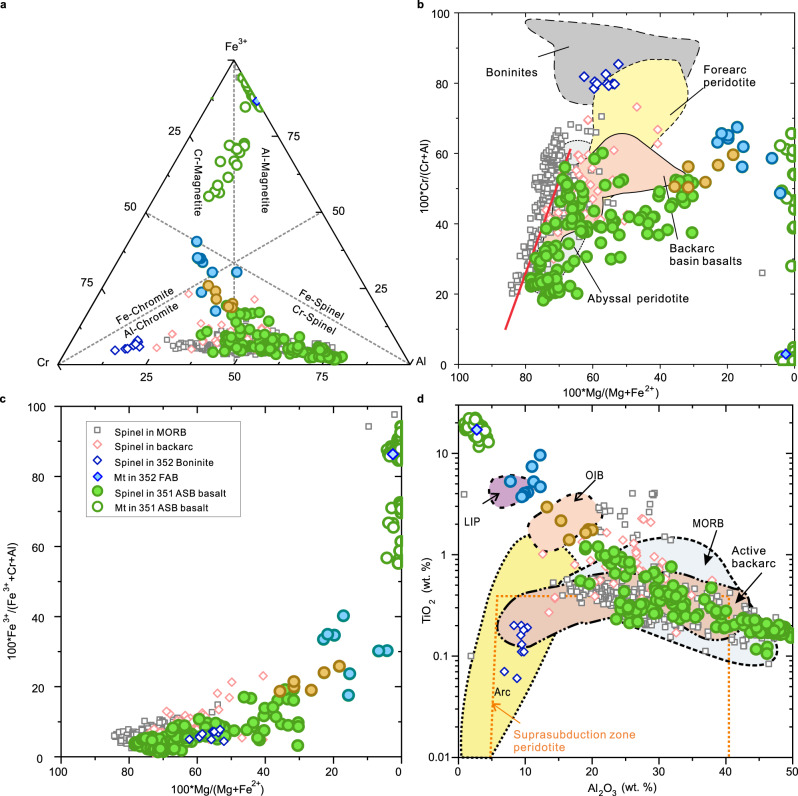
Spinel compositions in 351 Unit 1 ASB basalt compared with mid-ocean ridge basalts (MORB)^38,40^, 352 boninite^36^, backarc (data from PetDatabase website). **a** Cr-Al-Fe^3+^ cation diagram, dash lines are based on Stevens^66^. **b** 100∗Cr/(Cr + Al) versus 100∗Mg/(Mg + Fe^2+^). **c** 100∗Fe^3+^/(Fe^3+ ^+ Cr + Al)) versus 100∗Mg/(Mg + Fe^2+^). **d** TiO_2_ (wt. %) versus Al_2_O_3_ (wt.%). Green solid circles are spinel in Unit 1 and grey solid square symbols are spinel in MORB. Red line in (**b**) delimits the highest Mg/(Mg+Fe^2+^) for a given Cr/(Cr+Al) of the abyssal harzburgite array^41^. Coloured fields in (**b**) and (**d**) are based on Kamenetsky et al. (2001)^39^. To emphasise the compositional array, spinel compositions from Unit 1 that overlap the OIB (ocean island basalt) field are depicted with brown circles while those overlapping the LIP (large igneous province) field are shown with blue circles. The single published spinel (magnetite) analysis is from IBM FAB^36^.

The spinel in Unit 1 basalts is compositionally unusual on a global comparative basis^38,39^ Analyses are presented in Supplementary Table 4, and some representative photomicrographs are displayed in Fig. 2c–e. Grouped ternary and binary spinel compositional plots are shown in Fig. 4. Overall, the spinel in Unit 1 basalts has a unique compositional range compared with spinel in the most abundant basalt types of MOR, arcs, ocean islands and their possible progenitors, the large igneous provinces. Compositions form a continuum extending from Al-Cr-rich (pleonaste-chromite) to Ti-Fe^3+^-rich (titaniferous magnetite).

In the Al-Cr-rich range, Unit 1 spinel extends to more aluminous compositions than those of MORB^38,40^ (Fig. 4a), but have slightly lower Mg numbers (Fig. 4b, c). A notable feature is the Unit 1 spinel is consistently offset to lower Mg number for a given Cr/(Cr + Al) (Fig. 4b) and lower Fe^3+^/(Cr + Al + Fe^3+^) (Fig. 4c) than spinel in MORB. Both features are consistent with derivation from refractory peridotite sources and more reduced host magmas in the case of ASB basalt than for MORB^41,42^. In detail, the patterns of compositional zonation are complex, with spinel in the more evolved (lower Mg number) subunits b and c showing the most extensive and continuous zonation patterns (Supplementary Fig. 3). The general trend is characterised by relatively Al-rich cores zoned outwards through Cr- to Fe^3+^-rich compositions (Supplementary Fig. 3). Highly aluminous-spinels with the highest Al_2_O_3_ content (47 wt. %) in Unit 1 have the highest MgO contents (19.6 wt.%); these are higher than those in the majority of MORB, i.e. basaltic glasses from the Mid-Atlantic Ridge^40^.

Plagioclase is generally the most abundant mineral phase in Unit 1 ASB basalts. Analyses are presented in Supplementary Data Table 5. The range of compositions differs by subunit (Fig. 1c). In dry basalt magmas such as MORB, there is generally a consistent relationship between the forsterite contents of olivine with the anorthite contents of plagioclase (e.g., Fo_90_ in equilibrium with An_80_)^43^. In the case of comparatively wet arc basalts however, this correspondence is decoupled with the coexistence of markedly anorthitic plagioclase in equilibrium with less forsteritic olivine^44,45^. The Mg number of clinopyroxene in equilibrium with plagioclase mimics that of olivine. With the exclusion of subunit 1a wherein the plagioclase is a quench phase in the groundmass, the ranges in plagioclase composition are: b, An_88-52_; c, An_92-52_; d, An_74-56_; e, An_87-54_; f, An_85-68_. The decoupling of clinopyroxene Mg numbers and anorthite content of plagioclase is manifest in Fig. 1c. For example, the Mg numbers of clinopyroxene in Subunit 1d are consistently higher than the anorthite contents of the plagioclase; in contrast, much of the plagioclase in Subunit 1c is considerably more calcic than that of Subunit 1d, and considerably exceeds in numerical value the Mg number of the coexisting clinopyroxene. This type of pattern can be interpreted to reflect a wetter, evolved character (low Mg/(Mg + ΣFe)) of the host magma of 1c in contrast to the similarly evolved but relatively dry host of 1d. The fine-grained texture of 1d contrasts with the generally more phyric nature of 1c (Supplementary Fig. 1), and may reflect the relative dissolved water contents of the respective host subunit magmas.

### Trace elements of clinopyroxene

**Fig. 5:**
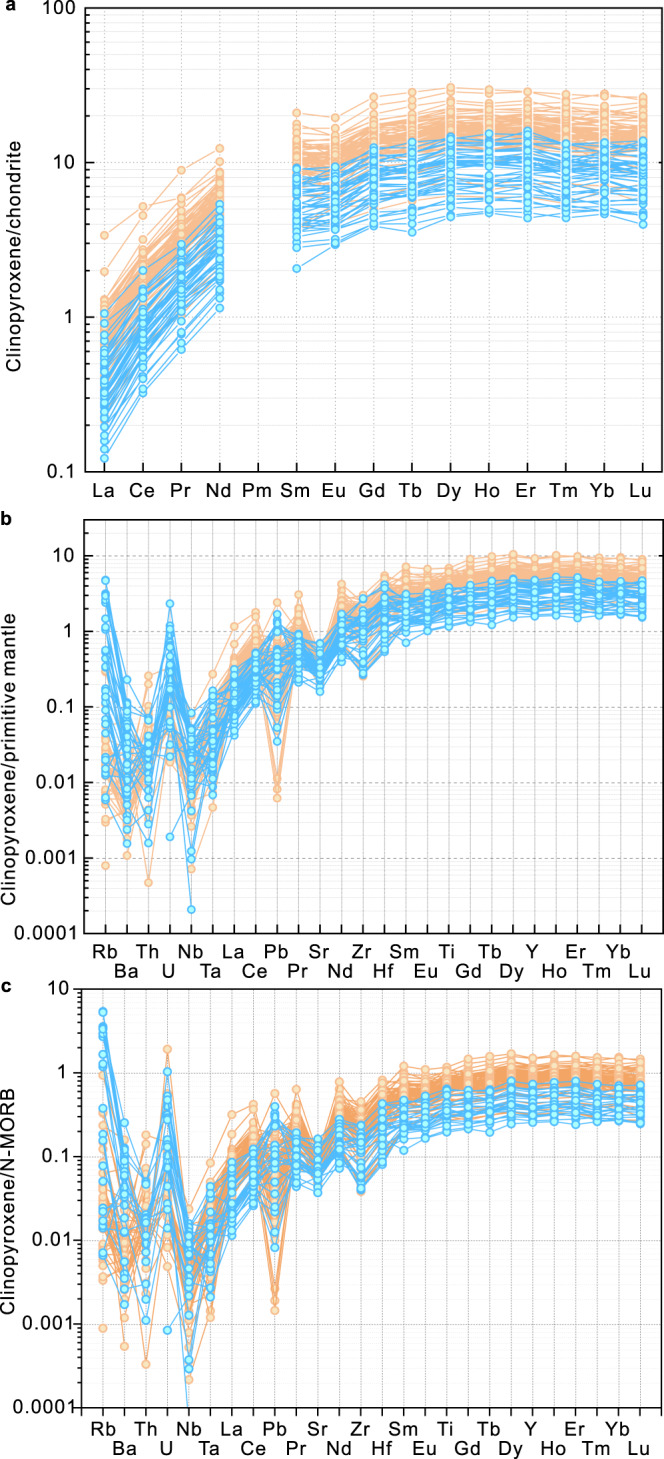
Trace element abundances for clinopyroxene. **a** Chondrite-normalized^47^ rare earth element abundances for clinopyroxene in subunits 1c and 1e of ASB basalts. **b** Primitive mantle-normalized^47^ trace element abundances for clinopyroxene in subunits 1c and 1e. **c** N-MORB-normalized^47^ trace element abundances for clinopyroxene in subunits 1c and 1e. N-MORB represents normal mid-ocean ridge basalt. Yellow circles are data for subunit 1c and blue-green circles for subunit 1e.

The trace element abundances of clinopyroxene are critically important for assessing the pristinity of the bulk trace element abundances of the Unit 1 ASB basalts. The partitioning of many trace elements, particularly the rare earths for example between clinopyroxene and basalt magma, is systematic and well understood^46^. Given the evidence for some seawater alteration of portions of the Unit 1 ASB basalts, the trace element abundances of the apparently pristine, unaltered clinopyroxene serve as an important monitor of the original trace element characteristics of the host magmas. The trace element abundances are presented in Supplementary Table 1. Chondrite-normalised REE and primitive mantle-normalised^47^ multi-element plots for representative high- and low- Mg/(Mg + ΣFe) subunits (e and c, respectively) are shown in Fig. 5. The general coupled variation of the Mg/(Mg + ΣFe) of the host basalt with Mg number of the clinopyroxene shown in Fig. 1c is sustained in the systematics of REE abundances. There is some overlap between clinopyroxene in subunits e and c (bulk rock high- and low-Mg/(Mg + ΣFe) respectively), but the abundances of all the REE in subunit 1e range to much lower values (e.g., La at 0.1* chondritic) than those of 1c, which conversely extend to much higher values (La at 3* chondritic) of all the REE. The order-of-magnitude range in abundances of a given rare earth for clinopyroxene in a specific subunit might relate to the open system behaviour of magma reservoirs tapped during eruption of Unit 1 basalts^48^. The trace element abundances of clinopyroxene in the other subunits are displayed in Supplementary Fig. 5; similar relationships between Mg/(Mg + ΣFe) of the subunit whole-rocks and the degrees of enrichment of the REE exist.

The crystallization order of plagioclase and clinopyroxene is related to the H_2_O content of the host melt^49^. Petrographic evidence can be ambiguous. Plagioclase has a greater preference for Eu^2+^ than clinopyroxene and the presence or otherwise of negative Eu anomalies in clinopyroxene is an indicator of prior plagioclase fractionation from a specific magma host. The overall range of Eu anomalies calculated using measured chondrite-normalised abundances of the specific elements in Eu/√(Sm∗Gd), is 1.2 to 0.6, with a mean value of expected/measured Eu = 0.88 (±0.09). Even the clinopyroxene with the highest Mg numbers in the most primitive subunits have this level of negative Eu anomaly; accordingly we infer saturation with plagioclase prior to clinopyroxene in all subunits of Unit 1.

The high abundances of potentially fluid-mobile trace elements (e.g., alkalis, alkaline earths, Pb and U) relative to immobile elements such as Nb and Zr, are a distinctive feature of arc magmas relative to MORB and OIB^50^. While none of these elements are strongly partitioned into clinopyroxene relative to coexisting melt, their abundances are nevertheless systematically related to those of a melt by the respective partition coefficients^46^. The primitive mantle-normalised abundances of these elements together with the REE in clinopyroxene of subunits e and c, have some distinctive characteristics: Rb and U are predominantly enriched relative to Nb, but Ba and Th are not (Fig. 5b). This decoupling is unusual. The abundance of Pb relative to Ce (nominally an element of similar peridotite-melt partitioning behaviour) is highly variable while Sr is generally depleted relative to Nd. These characteristics are typical of all clinopyroxene in Unit 1 (Supplementary Fig. 5). One interpretation of the decoupling is that Rb and U are elements typically enriched by circulation and reaction of seawater with basalts on the seafloor, whereas Th is not^51^. Elevated Unit 1 bulk-rock ^87^Sr/^86^Sr (0.7033-0.7060)^52^ is consistent with such a process. However, these patterns are present in apparently pristine clinopyroxene crystals with no signs of alteration.

### Trace element comparisons

**Fig. 6:**
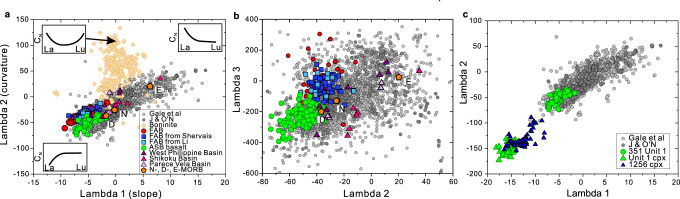
Shape coefficients of rare earth element patterns^53^ for comparison of Unit 1 ASB basalts with FAB, MORB/G, and backarc basin basalts of the Philippine Sea Plate. Data sources for a and b are Unit 1^26^, MORB from Gale et al.^28^ and Jenner & O’Neill^56^, boninite^20,67,68^, FAB of Izu-Bonin-Mariana^11,54,55^ and literature cited in ref. ^19^. **a** Lambda 2 (curvature) versus lambda 1 (slope). Schematic chondrite-normalised REE abundance patterns characterised variously by light REE-depletion, enrichment and U-shaped patterns of boninite are shown in the corners of this plot. N-, D-, E-MORB represent normal MORB, depleted MORB, and enriched MORB. **b** Lambda 3 (sigmoidal character) versus lambda 2. **c** Lambda 2 versus lambda 1 comparing clinopyroxene from Unit 1 and gabbros at Site 1256^69^ with ocean floor basalts^28,56^ and Unit 1 bulk rock^26^.

The systematic geochemical behaviour of the REE is of prime importance for understanding the processes of partial melting of the mantle, and fractional crystallisation of the resultant magmas. Quantification of the shapes of chondrite-normalised REE abundance patterns^53^ is particularly useful for systematically comparing the global array of ocean floor basalts with those of Unit 1 and FAB. In Fig. 6, the slope (lambda1), curvature (lambda 2), and extent of sigmoidal character (lambda 3) of respective patterns are displayed. The important features are Unit 1 basalts have the among most depleted and strongly downward-curved (−ve lambda 1 and 2, respectively) REE abundance patterns compared with MORB and other global ocean floor basalts, reflecting their derivation from a prior melt-depleted, spinel peridotite mantle source that was even more refractory than that tapped during the genesis of the vast majority of MORB (Fig. 6a). We emphasise the projections of slope and curvature of the chondrite-normalised REE abundances shown in Fig. 6 unequivocally reveal that Unit 1 ASB basalts are distinct compared with FAB of the Izu-Bonin-Mariana (IBM) arcs^11,54,55^ and backarc basin basalts of the Philippine Sea Plate. The latter is similarly depleted overall in REE but have less downward curvature. Furthermore, the sigmoidal character (lambda 3; upward curvature or flexure at the light REE end of otherwise concave-downward-sloping patterns) is also distinct between Unit 1 basalts of the ASB and FAB: the latter has a lesser degree of flexure (Fig. 6b).

Unit 1 ASB basalts are also distinctive when compared with the majority of active island arc basalts and boninites^53^. Most of the former overlap the MORB array in lambda 1 vs lambda 2 space, ranging upward from *λ1*, *λ2* of −5 and −40 respectively. Boninites plot in the upper centre of this type of diagram (*λ1* ~ 0; *λ2* > 25), reflecting their chondrite-normalised, concave-upward dish-shaped patterns^53^.

A comparison between the chondrite-normalised REE abundance patterns of the clinopyroxene in Unit 1 and the host rocks are shown in Fig. 6c. Those of the most primitive (highest 100∗Mg/(Mg + ΣFe)) subunits occupy the extremes of the respective clinopyroxene-bulk rock data arrays in negative lambda 1 vs 2 space. Fractional crystallisation of clinopyroxene drives the residual melt towards slightly more positive lambda values along the ocean floor basalt data array^53^.

## Discussion

The petrology and geochemical composition of Unit 1 ASB basalts provide an opportunity to probe the character of the source mantle extant at subduction inception, and what role the newly subducting Pacific Plate might have contributed to the earliest magma genesis. Compared with MORB and backarc basin basalt of the Philippine Sea, Unit 1 ASB basalts have high Mg/Fe, high Sc, low Ti, Zr, Ti/V, and Zr/Y^23^. They also have globally extreme, chondrite-normalised, depleted light REE abundance patterns^26^ (Fig. 6). The mineralogy of Unit 1 ASB basalts is also distinctive. A crystallisation sequence of spinel > olivine > plagioclase > clinopyroxene is inferred, similar to that of MORB. However, the high Mg number and strongly aluminous character of the clinopyroxene, and persistence until eruption of this phase coupled with the presence of spinel spanning a large Cr-Al-Fe compositional range, have not previously been identified in any ocean floor basalt.

Collectively, this evidence points to a refractory, albeit clinopyroxene-bearing, upper mantle source of Unit 1 ASB basalt, that had experienced a larger degree of prior melt extraction than that tapped during MORB generation^26,56^. The dry solidus temperature of this type of peridotite would be higher than that involved in MORB generation. Partial melting below the ASB nevertheless occurred ~3 million years after subduction inception, and was in the spinel peridotite stability field based on thermobarometry of the ASB whole rock (Supplementary Fig. 6). Contemporaneous tholeiitic basalts preceding boninites were being erupted in the Mariana forearc^57^. The presence of low-Fe^3+^, Cr-Al-rich spinel is a primary indicator of reduced conditions during source partial melting (Fig. 4).

Given the compositional zonation of the crystalline phases, and the uncertainty of establishing polyphase, heterogeneous equilibria, detailed thermobarometry (via a relevant reaction that includes spinel, olivine, plagioclase and pyroxene, such as 2Mg_2_SiO_4_ + CaAl_2_Si_2_O_8_ = MgAl_2_O_4_ + CaMgSi_2_O_6_ + Mg_2_Si_2_O_6_) is not feasible. There are however, two other approaches that can be used. One is based on the major element compositions of the most primitive (highest Mg#) basalts^55,58^, and the other uses single-phase thermobarometric algorithms, such as those applied to clinopyroxene-melt pairs in IBM FAB^36^. With the first approach, a range of extraction pressures of 0.6-2.1 GPa and temperatures between 1280–1470 °C were reported for Unit 1 ASB basalts^26^. If a range of primary MgO contents in Unit 1 ASB basalts from 12 to 13.5 wt% is correct, an alternate formulation^58^ gives a 10^5^ Pa (1 bar) potential temperature (*T*_p_) of 1350 to 1400°C for generation under dry conditions. These ranges are similar to those calculated for FAB generation^55^ (Supplementary Figure 6). Using single-phase thermobarometry for clinopyroxene in Unit 1 basalt with (FeO/MgO)_cpx_/(FeO/MgO)_melt_ = 0.26–0.27, gives 1090–1165 °C, at <0.6 GPa (Supplementary Figure 6 and Table 6). These results reflect crystallisation of the clinopyroxene during ascent of the host melts.

The reason for high temperatures for FAB was attributed to the addition of water and/or an effect of the Manus plume^55^. Several lines of evidence suggest Unit 1 ASB basalts contained dissolved H_2_O in excess of that typical of MORB, that would reduce these temperatures by ~40 °C for ~0.5 wt% dissolved H_2_O and be similar to the *T*_p_ of Mg-rich MORB^59^: (1) steepening of the clinopyroxene-in curve by dissolved H_2_O is a possible explanation for the preservation until eruption of this phase; (2) The high Mg number of the clinopyroxene is consistent with derivation of the parental Unit 1 ASB basalts directly from pressures (e.g., ~1–2 GPa) close to the transition from plagioclase- to spinel peridotite facies, where the difference in temperature of saturation between olivine and clinopyroxene is smaller than at lower pressures^49^; (3) contrasted degrees of crystallinity and variably anorthitic plagioclase is consistent with variable amounts of dissolved H_2_O; (4) A pressure-dependent upper limit (e.g., 2 wt% at 0.5 GPa) to dissolved H_2_O content is established by the persistence of saturation with plagioclase prior to clinopyroxene^49,59^. Elevated Rb and U relative to Ba and Th might be an intrinsic feature of unaltered Unit 1 ASB basalts; possibly Rb and U were introduced by dehydration at shallow (~30 km) depths of the newly descending Pacific Plate into the proto-mantle wedge of the IBM arc, that was still hot at shallow depths through involvement a few million years previously in the production of MORB along the Izanagi-Pacific Ridge^5^. Retention of Ba and Th in hydrous phases stable at relatively low pressures (e.g., mica, epidote, monazite) could account for the decoupling of these elements from Rb and U^60^.

Geochemical and mineralogical differences between the low-Ti-K tholeiitic basalt of the ASB and FAB may relate to the spatial and temporal evolution of the IBM system during the early stages of its development. The lower Ti/V of FAB^11,15,19,23^ might have resulted from the tapping of a similarly depleted peridotite source but with an oxidised overprint compared with that tapped during the generation of basalts in the ASB. For example, V is more incompatible in olivine-pyroxene-spinel assemblages at higher valence states. Consequently, Ti/V is a function of prior melt extraction, redox state during various melting episodes, and pressure/temperature of melting which is also related to H_2_O contents of the source peridotite^61,62^. For a given Zr concentration, the abundance of Cr in ASB basalt is generally higher than in FAB (Supplementary Fig. 7), possibly indicative of higher degrees of partial melting in the case of the former, assuming a common source composition.

The REE abundance patterns are not consistent with a more depleted source for FAB unless a light REE-bearing, subducted slab-derived component was added to the FAB source and not to the source of ASB basalts. The FAB is also generally less Mg-rich than Unit 1 ASB basalts, and the high-Mg, aluminous clinopyroxene plus Cr-Al-rich spinel assemblage characteristic of Unit 1 ASB basalt has not been observed in FAB. Eruption of the relatively unfractionated Unit 1 basalt requires rapid transit from the wedge sources without the prolonged staging characteristic of MOR systems^28,31^. An extensive rifting system within the overriding Philippine Sea Plate, both along- and across-strike of the nascent IBM subduction system is required, possibly due to rapid slab roll-back^7,63^. The near-trench transition to boninite magma generation required further partial melting of highly depleted mantle wedge sources but with a greater imprint of a slab-derived flux both of H_2_O and fluid-mobile trace elements^14,19^. The melt-depleted composition of this mantle wedge would have initially formed the sources tapped during the development of the KPR chain of stratovolcanoes, as sampled by Unit III pyroclastics^34^.

In conclusion, widespread development of magmatism in the IBM system following subduction initiation, both along- and across-strike, endured for at least ~3 Ma, prior to the development of the first stratovolcanoes. This development is marked by FAB (~52 Ma^19^) to concurrent boninite and ASB basalt (~49 Ma^24^) in the fore- and reararc respectively. The prior melt-depleted mantle source became increasingly refractory during this process. The ASB basalt, formed at the tipping point between FAB/boninite magmatism and typical arc development, is distinct compared to MORB, backarc basin, arc and OIB, and derived from highly refractory mantle sources. ASB basalt magma transferred rapidly from moderate pressures (~0.7–2 GPa) at the boundary between plagioclase- to spinel peridotite facies, to sub-crustal conditions. Vestiges of a polybaric-polythermal mineralogy are preserved in this basalt, and were not obliterated during the recharge-mix-tap-fractionate regimes typical of MOR or mature arcs.

## Methods

### Sample provenance and availability

All samples used in this study were taken from core material recovered by the IODP Expedition 351 at Site U1438. Residual (working-half) core material together with archival half-core is stored in the Kochi repository. Sample identification numbers used in this work are as mandated by the IODP.

### Scanning electron microscopy

Scanning electron microscopy in back-scattered electron imaging mode was used to characterize the morphology of the spinel in thin sections. Analysis was carried out at the Key Laboratory of Submarine Geosciences, State Oceanic Administration in China, using a JEOL 8100 probe, with an accelerating voltage of 15 kV, current of 10 nA and beam spot diameter of 1μm. Samples were coated with carbon prior to analysis.

### Electron probe micro-analysis

In situ major element compositions of minerals were obtained, both, using a Cameca SX-100 electron probe with four spectrometers at the Research School of Earth Sciences (RSES) in Australian National University (ANU), Canberra, Australian, and using JEOL-8200 electron probe at Guangzhou Institute of Geochemistry (GIG), Chinese Academy of Sciences (CAS). The operating conditions were: 15 kV of accelerating voltage, 20 nA of probe current, and 1 μm of the diameter of the electron beam. The counting times at the peaks were 10 s for Si, 20 s for Al and Mg, 30 s for Ti, Ca, Na and K, 40 s for Fe and Mn, and 60 s for Cr and P. Na, Mg, Al, Si and P were determined using Kα line obtained with a TAP crystal, then PET crystal for K, Ca, and Na, and LLIF crystal for Ni, Fe, Mn and Cr. Natural minerals were used as standards, and all data were corrected with a ZAF program.

### Laser ablation inductively coupled plasma-mass spectrometry (LA-ICPMS)

In situ trace element analyses of minerals were performed both at RSES in ANU, using Coherent CompexPro 110 laser ablation system connected to an Agilent 7700 ICP-MS, and at GIGCAS using a Resonetic Resolution S-155 laser ablation system connected on an Agilent 7900 ICP-MS. The laser spot size was set to 37 μm at ANU and 40 μm at GIGCAS. The analyzing were operated at a constant laser energy of 80 mJ, repetition rate of 6 Hz. The ablation time was 40 s with 30 s pre-ablation time and 20 s post-ablation time. NIST glass 612 was used as a standard and BCR-2G was used as a monitoring standard. The calculations of mineral trace element concentrations were performed by ICPMSDataCal 10.8^64^, using ^43^Ca as an internal standard. Replicate analyses result of trace elements of reference standard BCR-2G are summarized in Supplementary Table 7.

## Supplementary information

Supplementary information

## Data Availability

The authors declare that the data supporting the findings of this study are available within its supplementary tables in a Source Data file deposited at 10.6084/m9.figshare.13353074.v2. Backarc clinopyroxene and spinel data for comparison are downloaded from http://www.earthchem.org/petdb. Replicate analyses of trace elements of reference standard BCR-2G are compared to the GeoReM preferred values for each element, and recommended values are from http://georem.mpch-mainz.gwdg.de/. Figure 1a is made with GeoMapApp (www.geomapapp.org). Source data are provided with this paper.

## Source data

Source data
