# Supplementary material for: Basalt derived from highly refractory mantle sources during early Izu-Bonin-Mariana arc development
Source: Nat Commun. 2021 Mar 19;12:1723. doi: 10.1038/s41467-021-21980-0 (PMC7979767; doi:10.1038/s41467-021-21980-0)
Supplement: Supplementary file 1 — Supplementary information [file 41467_2021_21980_MOESM1_ESM.pdf]

Supplementary information

Basalt derived from highly refractory mantle sources during early Izu-Bonin-Mariana arc development

He Li[1,2,3,4*], Richard J. Arculus[4,*], Osamu Ishizuka[5], Rosemary Hickey-Vargas[6], Gene M. Yogodzinski[7], Anders McCarthy[8, 9], Yuki Kusano[5], Philipp A. Brandl[4,10], Ivan P. Savov[11], Frank J. Tepley III[12], Weidong Sun[1,2,3*].

[1]Center of Deep Sea Research, Institute of Oceanology, Chinese Academy of Sciences, Qingdao 266071, China. [2]Laboratory for Marine Mineral Resources, Qingdao National Laboratory for Marine Science and Technology, Qingdao 266237, China. [3]Center for Ocean Mega-Science, Chinese Academy of Sciences, Qingdao 266071, China. [4]Research School of Earth Sciences, Australian National University, Canberra, ACT 2601, Australia. [5]Geological Survey of Japan/AIST, Central 7 1-1-1 Higashi, Tsukuba, Ibaraki 305-8567, Japan. [6]Department of Earth & Environment, AHC5-394, Florida International University, Miami, FL 33199, USA. [7]Department of Earth & Ocean Sciences, University of South Carolina, Columbia, SC 29208, USA. [8]Institute of Earth Sciences, University of Lausanne, CH-1015 Lausanne, Switzerland. [9]School of Earth Sciences, University of Bristol, Wills Memorial Building, Queens Road, Clifton BS8 1RJ, UK. [10]GEOMAR Helmholtz Centre for Ocean Research Kiel, Wischhofstrasse 1-3, 24148 Kiel, Germany. [11]School of Earth & Environment, University of Leeds, Leeds LS2 9JT, UK. [12]College of Earth, Ocean, and Atmospheric Sciences, Oregon State University, Corvallis, OR 97331, USA.

[*]To whom correspondence should be addressed.

E-mail: lihe@qdio.ac.cn, ORCID number: 0000-0003-0363-0406

Richard.Arculus@anu.edu.au, ORCID number: 0000-0002-3432-392X

weidongsun@qdio.ac.cn, ORCID number: 0000-0002-9003-9608

Supplementary figures

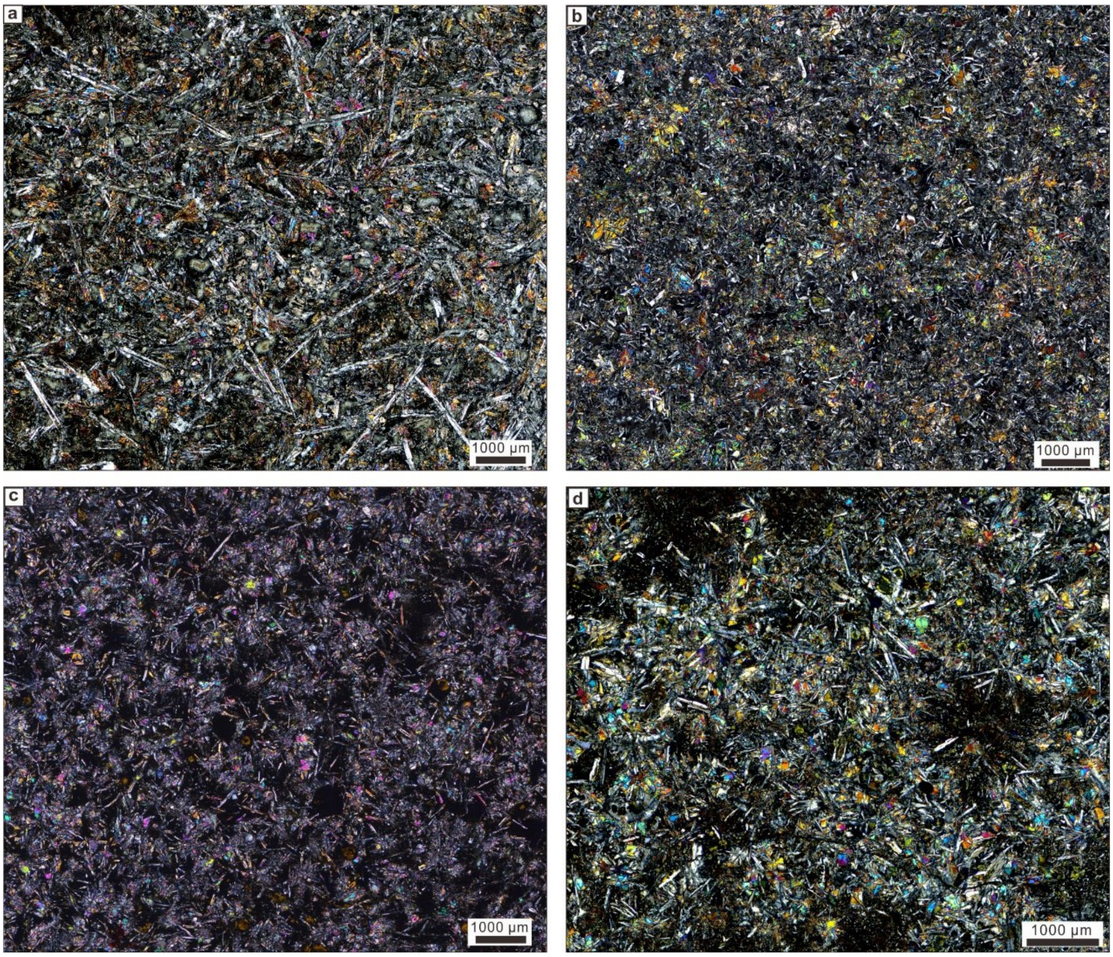

Supplementary Fig. 1. Cross-polarized images of ASB basalt from subunits 1a, 1b, 1d and 1f. (a) ASB basalt from subunit 1a (E71R2W90-94). **(b)** ASB basalt from subunit 1b (E72R1W83-85). **(c)** ASB basalt from subunit 1d (E81R1W54-57). **(d)** ASB basalt from subunit 1f (E88R1W139-145).

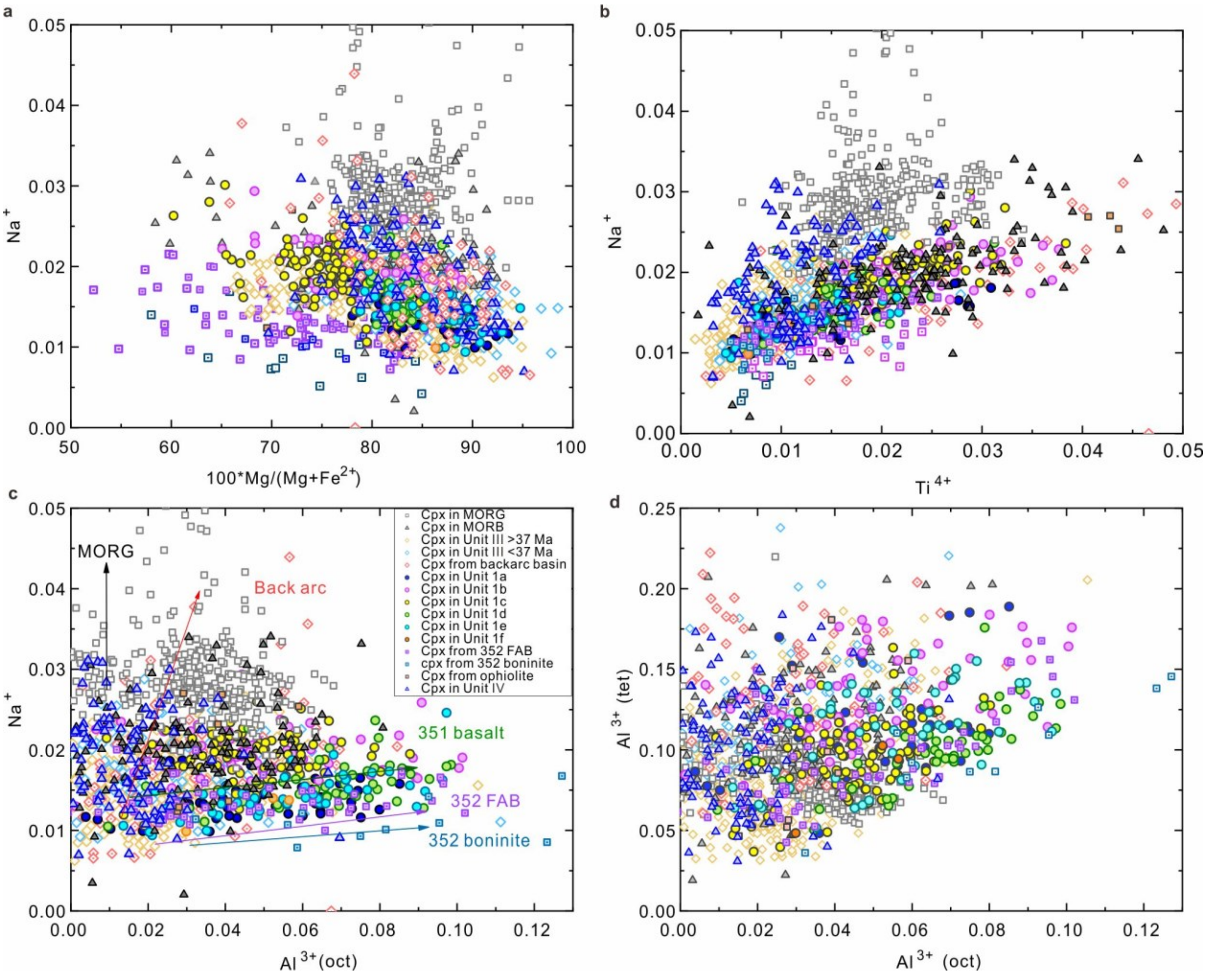

Supplementary Fig. 2. Clinopyroxene cations in subunits of Unit 1 ASB basalt compared with mid-ocean ridge basalt and gabbro (MORB/G)[1-10], Unit III, IV[11] ASB, backarc, ophiolite[12], and 352 forearc basalt (FAB) and boninite[13]. (a) Na^+ versus 100*Mg/(Mg+Fe^{2+}). Unit III is subdivided into 2 groups, respectively >37Ma and <37Ma, as identified by Brandl et al. (2017)[14], Unit IV data are from Waldman et al.[11]. With decreasing Mg number, Unit 1 clinopyroxene (solid circles) show a different trend of increasing Na compared with those of 352 FAB[13], backarc data from PetDatabase, and MORB/G[1-10]. Unit 1 clinopyroxene have higher Mg number than clinopyroxene in 352 FAB, especially subunit 1a and 1e. **(b)** Na^+ versus Ti^{4+}. A positive correlation between Na^+ and Ti^{4+} indicates no Na loss during the analytical process. **(c)** Na^+ versus octahedrally-coordinated Al^{3+}. Clinopyroxene in 352 FAB and boninite have low Na cations and high octahedrally-coordinated Al indicating their enrichment of Al and crystallization at relatively low temperature. Primitive clinopyroxene, i.e. subunit 1a and 1e, also have low Na cations and high octahedrally-coordinated Al, while subunit 1c have higher Na cations than other subunits at the same Al^{3+} (oct). **(d)** Tetrahedrally-coordinated versus octahedrally-coordinated Al^{3+} (Al^{3+} (tet) and Al^{3+} (oct) respectively). All cations calculated based on 4:6 cation:oxygen anion ratio.

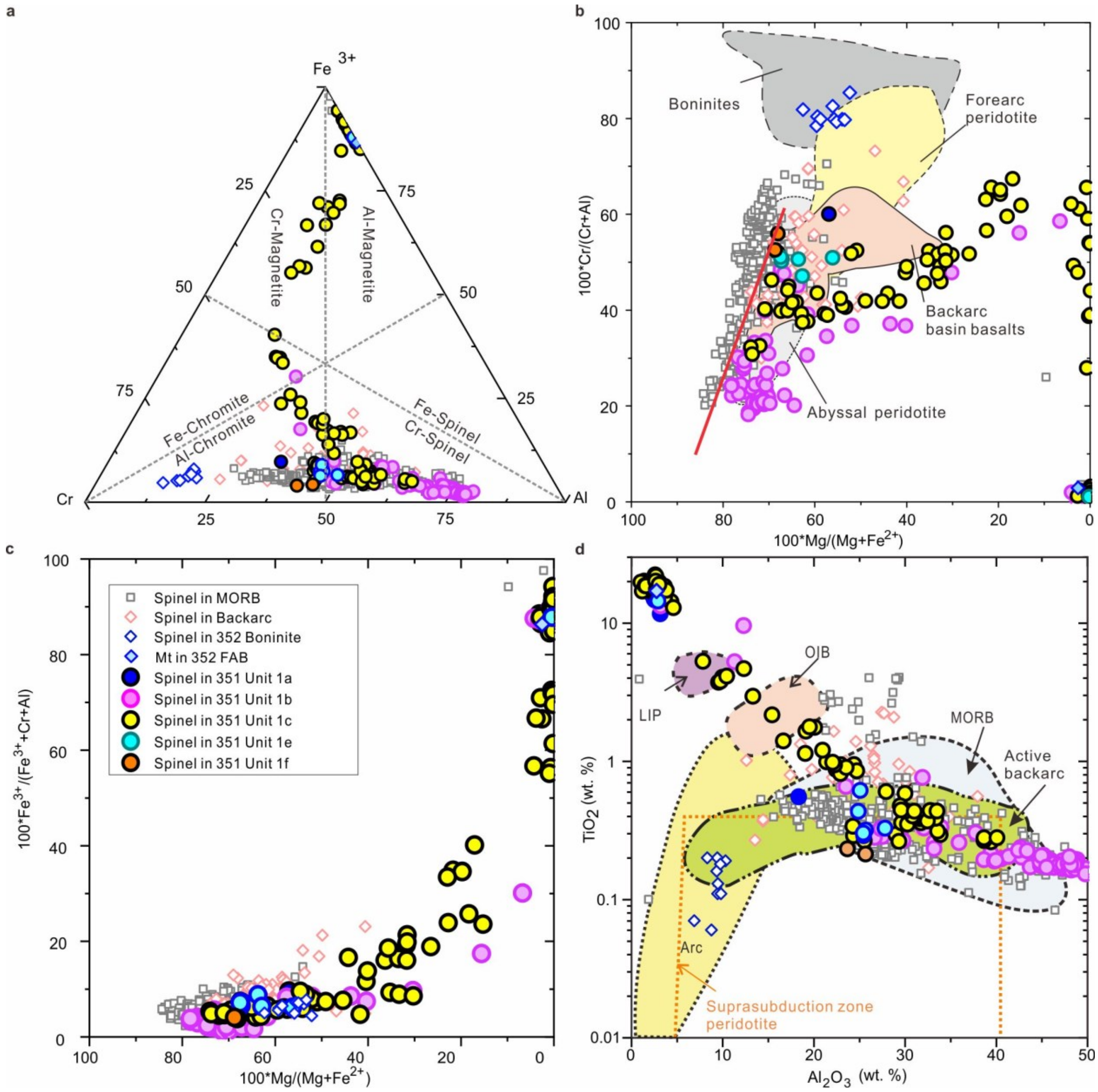

Supplementary Fig. 3. Spinel compositions in subunits of 351 Unit 1 ASB basalt compared with those in MORB[15-17], backarc (data from PetDatabase website), and 352 Boninite[13]. (a) Cr-Al-Fe^{3+} proportions. Dot lines are from Stevens[18]. Compositions of spinel in Unit 1 ASB basalt show unique compositional ranges, extending from Cr+Al-rich to Fe-rich, especially in subunit 1c. **(b)** 100*Cr/(Cr+Al) versus 100*Mg/(Mg+Fe^{2+}). Spinel in subunits 1b and 1c show different trends compared with those in backarc, forearc, and boninite. Offsets to lower Mg number for a given Cr/(Cr+Al) are consistent with derivation from refractory peridotite sources. **(c)** 100*Fe^{3+}/(Fe^{3+}+Cr+Al)) versus 100*Mg/(Mg+Fe^{2+}). Primitive spinel with high Mg number have low Fe^{3+}/(Fe^{3+}+Cr+Al) ratios indicating derivation from relatively reduced host magma. **(d)** TiO_2 (wt. %) versus Al_2O_3 (wt. %). Spinel in Unit 1 has higher TiO_2 and Al_2O_3 contents than those in forearc, ophiolite, and boninite, and have different covariation trends compared with those in backarc samples. Various coloured solid circles for spinel in respective subunits; the gray solid square symbols are for spinel in MORB. The single published spinel (magnetite) analysis is from IBM FAB[13].

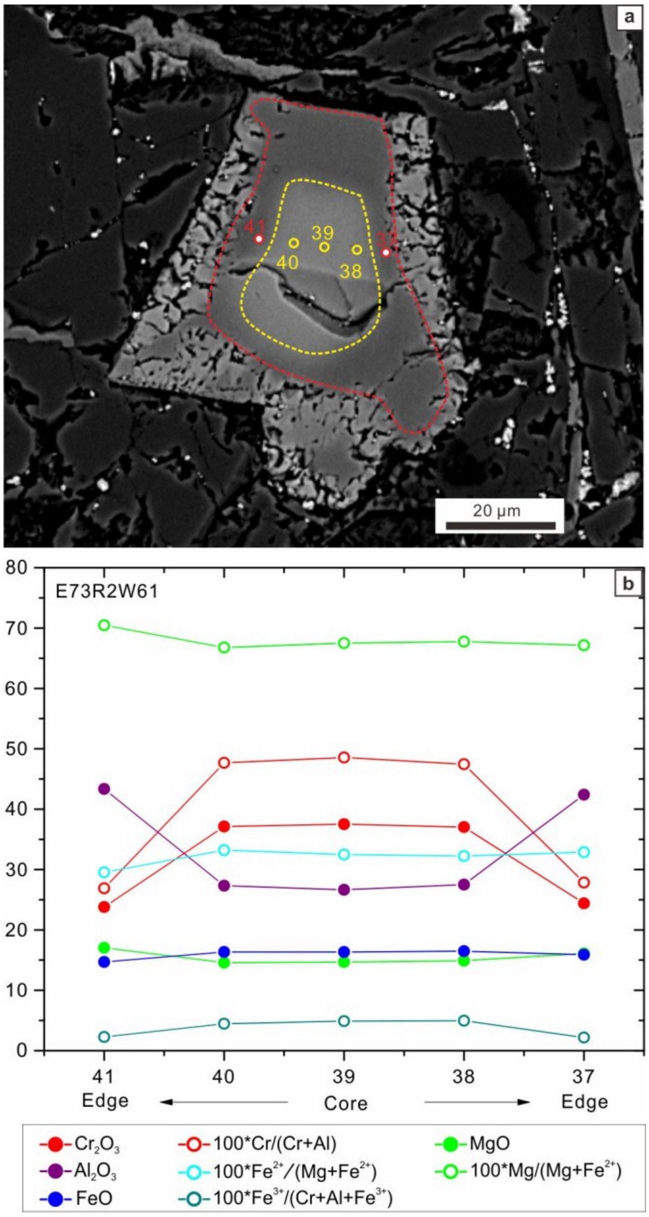

Supplementary Fig. 4. Back-scattered electron image and compositional traverse of spinel in subunit b sample 73R2 74-71. In this sample, compositions range from Cr-rich in the center of the exposed cross-section through Al-rich to magnetite on the rim. Generally, other 2D exposures reveal relatively Al-rich cores zoned outward through Cr-rich to Fe-rich compositions. Y-axis values are the oxide percentages or 100* specific cation ratios. Position numbers on the x-axis are keyed to the image.

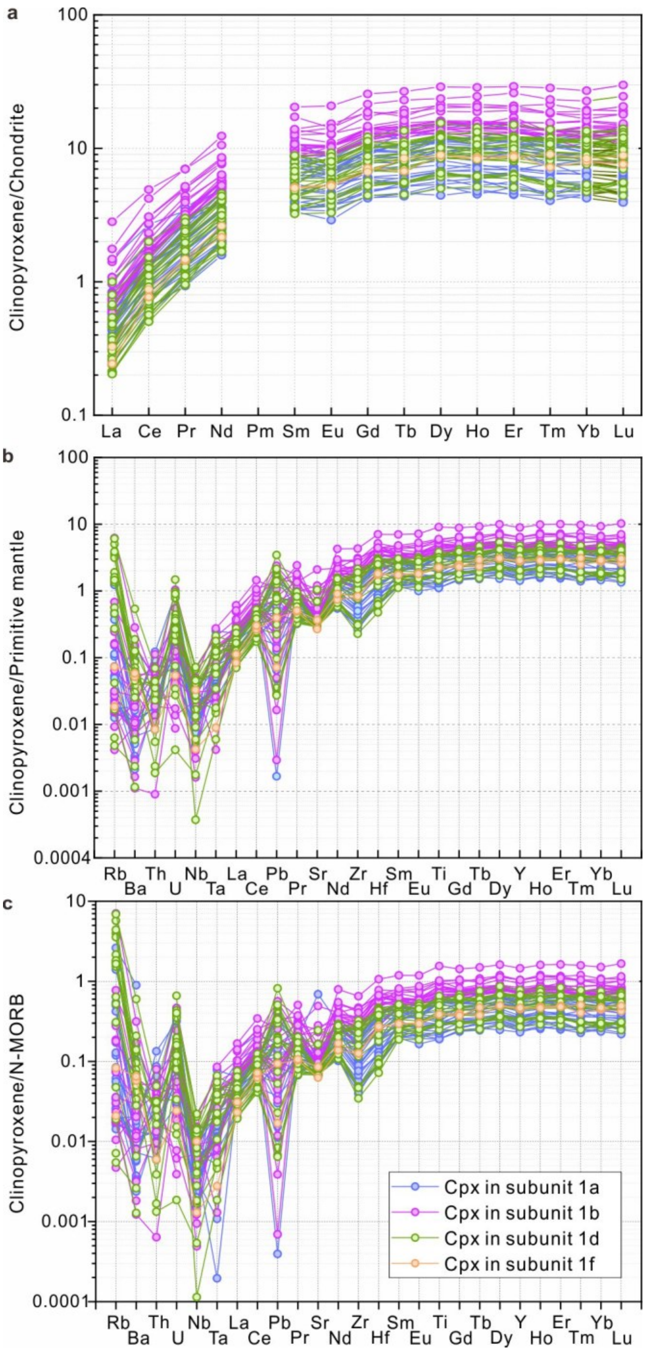

Supplementary Fig. 5. Trace element abundances for clinopyroxene in subunits a, b, d, and f. (a) chondrite-normalized[19] rare earth element abundances for clinopyroxene in subunits a, b, d, and f. **(b)** primitive mantle-normalized[19] trace element abundances for clinopyroxene in subunits a, b, d, and f. **(c)** N-MORB-normalized[19] trace element abundances for clinopyroxene in subunits a, b, d, and f. Clinopyroxene in all subunit have depleted light rare earth element (REE) and positive Rb, U anomalies. Subunit 1b has higher REE concentrations.

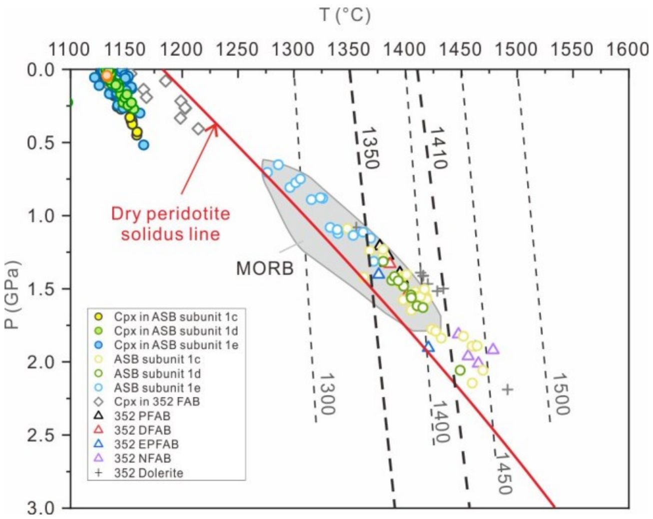

Supplementary Fig. 6. Pressure vs temperature conditions calculated for clinopyroxene-melt and melt-peridotite source equilbria[20], for ASB basalt and FAB[13, 20, 21]. Temperature and pressure equilibration conditions for clinopyroxene in ASB basalt were calculated based on Putirka et al. (1996)[22]. Dry peridotite solidus line and 1350ºC adiabat are from Sarafian (2017)[23]; the 1300, 1400, 1450, 1500 ºC adiabats and MORB field are from Shervais et al.[21].

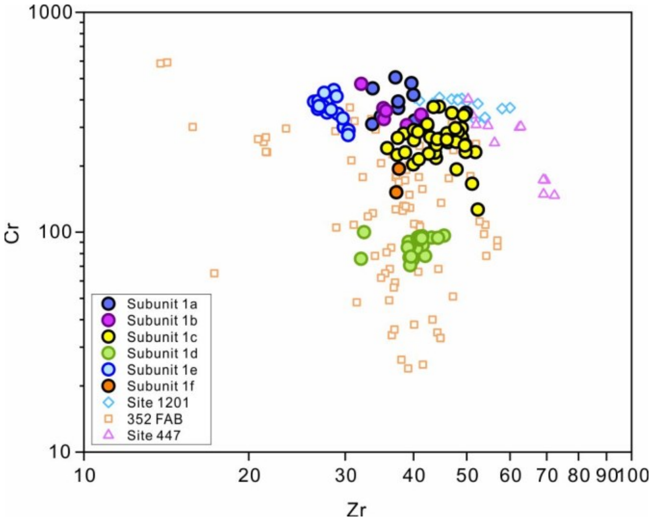

Supplementary Fig. 7. Cr vs. Zr diagram for whole rocks from 351 Unit 1 ASB basalt [20], 352 FAB [21, 24], Site 1201[25] and Site 447[25]. At the same Zr concentration, 351 Unit 1 basalt has higher Cr concentrations than FAB.

References for data sources used in construction of Fig. 3 and supplementary Fig. 2, 8 for clinopyroxene in MORB/G

MORB

1. Allan, J.F., Batiza, R. & Lonsdale, P.F. Petrology and chemistry of lavas from seamounts flanking the East Pacific Rise axis, 21 degrees N: implications concerning the mantle source composition for both seamount and adjacent EPR lavas. *AGU Geophys*. Mon. **43**, 255-282 (1987).
2. Batiza, R, Rosendahl, B.R. & Fishe, R.L. Evolution of oceanic crust 3. Petrology and chemistry of basalts from the East Pacific Rise and the Siqueiros Transform fault. J. *Geophys. Res.* **82**, 265-276 (1977).
3. Batiza, R. et al. Petrology, geochemistry, and petrogenesis of Leg 142 basalts – synthesis of results. *Proc. ODP Sci. Res*. **142**, 3-8 (1995).
4. Griffin, B.J., Neuser, R.D. & Schmicke, H.U. Lithology, petrography, and mineralogy of basalts from DSDP sites 482, 483, 484, and 485 at the mouth of the Gulf of California. *Initial Rep. DSDP* **65**, 527-548 (1983).
5. Hekinian, R. & Walker, D. Diversity and spatial zonation of volcanic rocks from the East Pacific Rise near 21 degrees N. *Contrib. Mineral. Petrol*. **96**, 265-280 (1987).
6. Hekinian, R. et al. Offset spreading centers near 12o 53'N on the East Pacific Rise: submersible observations and compositions of the volcanics. *Mar. Geophys. Res*. **7**, 359-377 (1985).
7. Rowbotham, G. & Floyd, P.A. Mineral chemistry of primary and secondary phases in basaltic rocks, Leg 129. *Proc. Ocean Drilling Prog., Sci. Results* **129**, 305-343 (1992).
8. Thompson, R.N. & Humphris, S.E. Silicate mineralogy of basalts from the East Pacific Rise, OCP Ridge, and Siqueiros Fracture Zone: Deep Sea Drilling Project Leg 54. *Initial Rep*. DSDP **54**, 651-669 (1980).

MORG

9. Dick, H.J.B. et al. Primary silicate mineral chemistry of a 1.5-km section of very slow spreading lower ocean crust: ODP Hole 735B, Southwest Indian Ridge. *Proc. ODP Sci. Res*. **176**, 1-61(2002).

10. Fujibayasi, N., Kagami, H. & Oishi, Y. Mineralogy and Sr- and Nd-isotopic composition of gabbroic oceanic crust recovered from holes 923A and 921E in the MARK area. In Karson, J.A., Cannat, M., Miller, D.J., & Elthon, D. (eds) *Proc.Ocean Drill. Prog., Sci. Res*. **153,** 471-490 (1997).

11. Waldman R. J., et al. Sedimentary and volcanic record of the nascent Izu-Bonin-Mariana arc from IODP Site U1438. *GSA Bulletin*, **doi: 10.1130/b35612.1** (2020).
12. Alabaster T., Pearce J. A., Malpas J. The volcanic stratigraphy and petrogenesis of the Oman Ophiolite complex. *Contributions to Mineralogy and Petrology* **81**, 18-183 (1982).
13. Whattam S. A., et al. Mineral compositions and thermobarometry of basalts and boninites recovered during IODP Expedition 352 to the Bonin forearc. *American Mineralogist* **105**, 1490-1507 (2020).
14. Brandl P. A., et al. The arc arises: The links between volcanic output, arc evolution and melt composition. *Earth and Planetary Science Letters* **461**, 73-84 (2017).
15. Barnes S. J., Roeder P. L. The range of spinel compositions in terrestrial mafic and ultramafic rocks. *Journal of Petrology* **42**, 2279-2302 (2001).
16. Kamenetsky V. S., Crawford A. J., Meffre S. Factors controlling chemistry of magmatic spinel: An empirical study of associated olivine, Cr-spinel and melt inclusions from primitive rocks. *Journal of Petrology* **42**, 655-671 (2001).
17. Sigurdsson H., Schilling J. G. Spinels in Mid-Atlantic Ridge basalts: Chemistry and occurrence. *Earth and Planetary Science Letters* **29**, 7-20 (1976).
18. Stevens R. E. Compositions of some chromites of the western hemisphere. *American Mineralogist* **29**, 1-34 (1944).
19. Sun S. S., McDonough W. F. Chemical and isotopic systematics of oceanic basalts: Implications for mantle composition and processes. In Saunders, AD, & Norry, MJ (eds) Magmatism in the Ocean Basins, *Geological Society London Special Publications* **42**, 313-345 (1989).
20. Hickey-Vargas R., et al. Origin of depleted basalts during subduction initiation and early development of the Izu-Bonin-Mariana island arc: Evidence from IODP expedition 351 site U1438, Amami-Sankaku basin. *Geochimica et Cosmochimica Acta* **229**, 85-111 (2018).
21. Shervais J. W., et al. Magmatic Response to Subduction Initiation: Part 1. Fore-arc Basalts of the Izu-Bonin Arc From IODP Expedition 352. *Geochemistry Geophysics Geosystems* **20**, 314-338 (2019).

22. Putirka K., Johnson M., Kinzler R., Longhi J., Walker D. Thermobarometry of mafic igneous rocks based on clinopyroxene-liquid equilibria, 0-30 kbar. *Contributions to Mineralogy and Petrology* **123**, 92-108 (1996).
23. Sarafian E., Gaetani G. A., Hauri E. H., Sarafian A. R. Experimental constraints on the damp peridotite solidus and oceanic mantle potential temperatur. *Science* **355**, 942-945 (2017).
24. Li H.-Y., et al. Radiogenic isotopes document the start of subduction in the Western Pacific. *Earth and Planetary Science Letters* **518**, 197-210 (2019).
25. Yogodzinski G. M., et al. Implications of Eocene-age Philippine Sea and forearc basalts for initiation and early history of the Izu-Bonin-Mariana arc. *Geochimica et Cosmochimica Acta* **228**, 136-156 (2018).
